# A Review on Antistaphylococcal Secondary Metabolites from Basidiomycetes

**DOI:** 10.3390/molecules25245848

**Published:** 2020-12-11

**Authors:** Vimalah Vallavan, Getha Krishnasamy, Noraziah Mohamad Zin, Mazlyzam Abdul Latif

**Affiliations:** 1 Center for Diagnostic, Therapeutics & Investigative Studies, Faculty of Health Sciences, Universiti Kebangsaan Malaysia, Jalan Raja Muda Abdul Aziz, Kuala Lumpur 50300, Malaysia; p99918@siswa.ukm.edu.my (V.V.); noraziah.zin@ukm.edu.my (N.M.Z.); 2 Bioactivity Program, Natural Products Division, Forest Research Institute Malaysia (FRIM), Kepong 52109, Selangor, Malaysia; 3 Center for Toxicology and Health Risk Studies, Faculty of Health Sciences, Universiti Kebangsaan Malaysia, Jalan Raja Muda Abdul Aziz, Kuala Lumpur 50300, Malaysia; mazlyzam@ukm.edu.my

**Keywords:** Basidiomycota, bioactive natural products, antibacterial, antimicrobial resistance, methicillin-resistant *Staphylococcus aureus* (MRSA)

## Abstract

Fungi are a rich source of secondary metabolites with several pharmacological activities such as antifungal, antioxidant, antibacterial and anticancer to name a few. Due to the large number of diverse structured chemical compounds they produce, fungi from the phyla Ascomycota, Basidiomycota and Muccoromycota have been intensively studied for isolation of bioactive compounds. Basidiomycetes-derived secondary metabolites are known as a promising source of antibacterial compounds with activity against Gram-positive bacteria. The continued emergence of antimicrobial resistance (AMR) poses a major challenge to patient health as it leads to higher morbidity and mortality, higher hospital-stay duration and substantial economic burden in global healthcare sector. One of the key culprits for AMR crisis is *Staphylococcus aureus* causing community-acquired infections as the pathogen develops resistance towards multiple antibiotics. The recent emergence of community strains of *S. aureus* harbouring methicillin-resistant (MRSA), vancomycin-intermediate (VISA) and vancomycin-resistant (VRSA) genes associated with increased virulence is challenging. Despite the few significant developments in antibiotic research, successful MRSA therapeutic options are still needed to reduce the use of scanty and expensive second-line treatments. This paper provides an overview of findings from various studies on antibacterial secondary metabolites from basidiomycetes, with a special focus on antistaphylococcal activity.

## 1. Introduction

Antimicrobial resistance (AMR) crisis is associated with more than 2 million hard-to-treat infectious diseases. The Center for Disease Control and Prevention (CDC) reported that increasing mortality rate at an average of 23,000 deaths per year was recorded in developing countries [1]. Major pathogen that contributes to the AMR incidence is *Staphylococcus aureus* with the emergence of multidrug-resistant strains such as methicilin-resistant (MRSA), vancomycin-intermediate (VISA) and vancomycin-resistant (VRSA) *S. aureus* [2,3,4]. The rising incidence of these resistant pathogens leads to inadequate antimicrobial therapeutic effects that are related to poor healthcare outcome in patients. Community-acquired methicillin-resistant *S. aureus* (CA-MRSA) strains also account for an increasing proportion of hospital-acquired MRSA (HA-MRSA) infections [3,5]. These pathogenic strains of *Staphylococci* with their intrinsic virulence factor can cause a diverse array of life-threatening infections [4]. The high antibiotic selective pressure in crowded populations, like in Asia, creates an environment that allows rapid development and successful spread of multidrug-resistant pathogens such as HA-MRSA and CA-MRSA [3,6]. The last resort treatment for MRSA infections is vancomycin [2]. However, current loss in sensitivity toward vancomycin limits the conventional therapeutic choice for *Staphylococcal* infections [2,7].

Fungal secondary metabolites have been reported as a potential source of bioactive compounds with antibacterial activity. The accidental discovery of penicillin from fungi in 1929 by Fleming drew attention of scientific community to the possible role of fungi as antibiotics and this has contributed to the isolation and development of other antibiotics [8]. Fungi are rich sources of secondary metabolites with diverse bioactivities, many that have been developed into important pharmaceutical products. With more than 15,000 secondary metabolites discovered to date, fungi stand out as an important group of microbes in bioactive natural products research [9]. Advances in analytical chemistry, computational tools, and drug discovery research have enabled the development of some fungal-derived antimicrobial compounds with potential therapeutic effects to be used individually or in adjunctive therapies to control difficult-to-treat pathogens [10]. Secondary metabolites from saprotrophic and easily cultivable fungi of the phyla Ascomycota, Muccoromycota and Basidiomycota have been studied intensively [11].

Previous studies have shown that secondary metabolites from basidiomycetes have a wide range of pharmacological activities including antimicrobials [12]. Basidiomycetes, from the phylum Basidiomycota, are a group of higher fungi with distinctive fruiting bodies and reproductive structures with edible and non-edible properties. Mushroom-forming fungi, mostly from the basidiomycete group, have been used as remedies for various diseases owing to their ability to produce compounds with high structural diversity, including terpenes, anthraquinone, derivatives of benzoic acid, quinolines, cyclic peptides, steroids, sesquiterpenes, oxalic acid, epipolythiopiperazine-2,5 diones and polysaccharides [13,14]. Traditionally, bioactive components have been extracted from fruiting bodies or mycelial extracts of mushrooms [15]. They are known to produce secondary metabolites with a range of pharmacological activities including antimicrobial, antioxidant, anti-angiogenesis, anticancer, immunomodulatory and anti-inflammatory [16].

In many studies, however, antimicrobial activities of different extracts of mushroom were reported without identifying the active compound/s responsible for the observed high activity against Gram-positive bacteria [13,17,18]. Despite the challenges faced in explorative studies to access the bioactive metabolites originating from fruiting bodies of mushrooms as they occur temporarily in the environment, their importance has been significant in recent decades [13]. With regards to this, more studies have been focusing on metabolites produced from submerged fermentation of mycelial culture of mushrooms where frequently these metabolites differ from those of fruiting bodies [19]. This work is a brief review on antistaphylococcal activities of Basidiomycetes that have been reported.

### 1.1. Antimicrobial Resistance (AMR)

Antimicrobial resistance is described as lowered efficiency or loss of antibiotics’ effectiveness against pathogens and this is a major problem in the medical sector globally. Antimicrobial resistance is correlated with high medical costs because of a longer period of disease, additional testing and needless usage of second-line treatments [1,5]. As mentioned by the Organization for Economic Co-operation and Development (OECD), the key risk factor for development of resistance is excessive usage or intake of antibiotics [6,20,21]. The high emergence of AMR has led to a shift change in therapeutic practices towards use of newer wide-spectrum drugs and increased usage (42%) of last resort classes of antibiotics such as vancomycin [6]. Many reports have indicated that the resistance epidemiology is global and spreads through nations and across borders [20].

In vitro antibacterial activity of antibiotics is typically determined by biological assays. The most popular methods include agar well diffusion, disc diffusion, minimum inhibitory concentration (MIC), minimum bactericidal concentration (MBC) and time kill assays [22]. Clinical breakpoints used as interpretive criteria to consider susceptibility of a bacterial isolate to an antimicrobial agent are provided by the Clinical and Laboratory Standards Institute (CLSI) [23,24], the European Committee on Antimicrobial Susceptibility Testing (EUCAST) and/or the US Food and Drug Administration (FDA).

In agar well diffusion assay, a hole was punched into agar inoculated with the test organism and filled with the antibiotic solution. Alternatively, a filter paper disc containing antibiotic was placed on inoculated agar. In both methods, zone of inhibition produced by the diffusion of antibiotic compound into the agar was measured. Due to the agar being an aqueous preparation, non-polar compounds do not diffuse as well as polar compounds, thus producing smaller diameter of inhibition zones despite their higher activity. This could be a limitation in the agar diffusion method [25,26]. The MIC is defined as the lowest concentration of a drug that inhibits the growth of bacteria after incubation. The MBC of a drug is determined upon reading of MIC by streaking the broth dilutions onto general or selective agar with 24–48 h of incubation. Absence of growth of the viable organisms on agar indicated the lowest broth dilution of drug, which caused a 99.9% suppression of the bacterial growth. The most appropriate in vitro approach to study bactericidal activity of a vast variety of antimicrobial agents is the time-kill assay [22]. The outcome of this assay indicates if an antimicrobial effect is dependent on exposure time or concentration of the drug. The assay is often used as initial descriptive analysis in pharmacodynamic analysis of a drug [22,27].

Preventing the development of resistant bacterial strains is important to ensure the effectiveness of current drugs in managing dangerous and life-threatening infections as an attempt to reduce the severity of AMR crisis [28,29]. Thus, there is an urgent need to carry out continuous research and development of new antibacterial drugs to counter the loss in efficacy of current antibiotics [28,30,31].

### 1.2. Multidrug Resistance in Staphylococcus aureus

*Staphylococcus aureus* infections produce wide spectrum of pyogenic lesions involving several organs, and it can cause hospital outbreaks and community acquired infections. Selective pressure on the bacteria due to high consumption of wide-spectrum antibiotics could stimulate the emergence of antibacterial resistant strains. Burden of infections in low-income countries is high since the solution to overcome this crisis is by replacing ineffective first line antibiotics to more costly second line or third line antibiotics [6]. Thus, the development of new antibiotics, combination drugs, bioprospecting for potential antibacterial natural compounds and improved drug delivery systems are some of the current strategies to control the antimicrobial resistance threat [6,32].

Emergence of HA-MRSA strains are associated with profligate use of antibiotics in healthcare settings [33]. The MRSA strains have demonstrated resistance to a range of antibiotics belonging to isoxazoyl penicillin group (methicillin, oxacillin, flucloxacillin), cephalosporins and carbapenems [34,35,36]. The first MRSA variant strain was isolated in United Kingdom in 1961 after methicillin was introduced in 1959 [37,38,39,40]. Thereafter, the changing epidemiology of variants of the strains found in many other countries like Europe, Australia, Japan and United States eventually makes MRSA as a major threat in nosocomial infections worldwide [20]. MRSA has more propensity to develop resistance to macrolides, quinolones and aminogylycosides, and this led to reduced therapeutic options [34,35,36,41,42]. In hospitals worldwide, a high prevalence of MRSA with rates above 50% has been documented [43,44]. A new variant strain of CA-MRSA was reported to be prevalent in Asian healthcare settings. This was documented by several studies which showed an occurrence rate of 2.5% in Thailand and 38.8% in Sri Lanka [45].

Emergence of antimicrobial resistance in *S. aureus* to glycopeptide group of antibiotics which is the last resort of staphylococcal treatment, became a global concern in managing staphylococcal infections [29]. Three classes of limited vancomycin susceptibility strains of *S. aureus* that have emerged in different locations around the world are VISA, heterogeneous VISA (hVISA) and VRSA [37,46]. Owing to the dynamic re-organisation of cell wall metabolism, VISA and hVISA strains have thickened cell walls with decreased glycopeptide cross-linking [44]. The first report of VISA and hVISA was detected in Japan in 1996 and 1997, respectively, while VRSA from a hospital in the United States was reported in 2002 [47]. The resistance phenotypic of VISA (Minimum Inhibitory Concentration: 8 µg/mL) has the ability of reverting back to the susceptibility phenotype towards vancomycin when the selective pressure is removed (MIC at 2 µg/mL) [48].

Prevalence of VRSA strains have been documented in South Nigeria (0–6%), Zaria, North Nigeria (57.7%), South India (1.4%), Australia, South Africa, Scotland, Hong Kong, Thailand and Korea (0–74%) [39,48,49,50]. No reports of vancomycin-resistant *S. aureus* (VRSA) have been documented in Malaysia [51]. The emergence of antibiotic resistance globally could lead to serious problems of limited therapeutic options available [52]. The emergence of VISA and VRSA strains causes more life-threatening infections in the healthcare sector [53,54]. Scanty and expensive drugs like teicoplanin, daptomycin and linezolid are also being used as next therapeutic options for MRSA infection due to the limited sensitivity of vancomycin [15,35,54,55,56].

## 2. Anti-MRSA Drug Discovery from Fungi

Research on the development of novel drugs from natural products is faced with limitations and obstacles since pharmaceutical companies earn low profits from this sector. However, screening of natural products for new bioactive compounds in discovery programmes hold high prospects to slow down the development of antimicrobial resistance crisis. Penicillin, which was discovered from *Penicillium notatum*, had been used to derive some commercial antibiotics that acts against Gram-positive bacteria deep infections such as Piperacillin, Amoxicillin and Ampicillin [9,57,58,59].

Cephalosporin C, which belongs to the class of β-lactam antibiotics, was developed from *Cephalosporium acremonium* and the commercialised products are Cephalexin, Cephradine and Cefadroxil [9]. The β-lactam antibiotics are active against Gram-positive bacteria including *S. aureus*. Fusidic acid, the steroid-like topical antibiotics that was produced from *Fusidium coccineum* or *Acremonium fusidioides,* showed antistaphylococcal activity against penicillin and methicillin-resistant strains of *S. aureus* [60,61]. The commercialised products from tripene structured fusidic acid metabolite are Usidin, Fucidin, Fucicort, Fucibet and Taksta [9]. Retapamulin (Altabax) is a derivative from the secondary metabolite pleuromutilin produced from *Pleurotus mutilis* (Fr.) Sacc. and *Pleurotus passeckerianus* Pilat (Pleurotaceae). Pleuromutilin which was discovered from Basidiomycete origin, has been used to treat infections induced by Gram-positive bacteria including MRSA [62,63].

Glycopeptides such as the semi-synthetic oritavancin are broad spectrum antibiotics against Gram-positive bacteria including MRSA and VRSA. The bacteria-derived compound teixobactin, has potential antistaphyloccoccal activity with MIC value of 0.25 µg/mL and is comparatively superior to vancomycin in killing late exponential phase populations of the pathogen and bactericidal activity against VISA [58].

## 3. Antistaphylococcal Natural Products from Basidiomycetes

Basidiomycetes derived secondary metabolites are known as a promising source of antibacterial compounds with activity against Gram-positive bacteria in natural product discovery. Crude extracts of natural products were reported to target on cell wall biosynthesis and cell membrane permeability as their mechanism of action to exhibit antibacterial activity [64]. Many species have been studied for their potential to produce bioactive secondary metabolites with antibacterial activity against *S.aureus* and other drug resistant strains of bacteria as shown in Tables S1–S3.

The fruiting body extracts of *Ganoderma* species such as *G. applanatum* have shown positive antistaphylococcal activity [65,66]. Quereshi et al. [67] showed that metabolites derived from *Ganoderma* mushroom possessed bacteriolytic enzyme, lysozyme, acid protease and polysaccharides as bioactive principles that play a role in antibacterial activity [67]. The most common species that has been explored for pharmaceutical activity is *G. lucidum* [68,69]. *Ganoderma lucidum* has been regarded as a potential producer of broad-spectrum antibacterial compounds highly potent against Gram-positive bacteria, as indicated by preclinical (in vitro and in vivo animal) studies [69]. *Ganoderma lucidum* is saprobic in nature and secondary metabolites have been extracted from the fruiting body using methanol extraction. The antistaphylococcal activity of extracts from fruiting body of *G. lucidum* strain was represented by inhibition zone diameter ranging from 2.2 to 2.5 cm recorded against *S. aureus* [15]. The isolated compound, 12β-acetoxy-3β, 7β-dihydroxy-11, 15, 23-trioxolanost-8-en-26-oic acid butyl ester (**1**) from *G. lucidum* displayed MIC value of 68.5 um against *S. aureus* [70]. *Ganoderma pfeifferi* is one of the phytochemically less investigated species of the family Ganodermataceae. *Ganoderma pfeifferi* produced farnesyl hydroquinones, namely ganomycins A (**2**) and B (**3**), which are potential inhibitors of *S. aureus* and MRSA [19,71]. The lack of data on mechanism of action of the antimicrobial compounds from *Ganoderma* spp. does not support their use as a sole antibiotic. Further studies are needed before these compounds are made available for human use [69].

The fruiting bodies of *Tapinella atrotomentosa* were extracted with methanol and the fractionated compounds were isolated using High Performance Liquid Chromatography analysis. Compounds from *T. atrotomentosa* include osmundalatone (**4**), 5-hydroxy-hex-2-en-4-olide (**5**) and spiromentin C (**6**), which showed positive growth inhibiting activity against MRSA (SZMC 6270) at higher MIC value of 250 µg/mL. Another compound from this fungus, spiromentin B, showed no activity against MRSA. Moreover, the interaction between cefuroxime and the isolated compounds was evaluated against MRSA. Results indicated that the compounds do not act as an adjuvant to increase the antibiotic activity. The antioxidant activity using 2,2-diphenyl-1-picryl-hydrazyl-hydrate(DPPH) and Oxygen Radical Absorbance Capacity (ORAC) assays showed that spiromentin C (**6**) and B exhibited high antioxidant effects (16.21 ± 0.38 and 11.23 ± 0.58 mmol TE/g, respectively) compared to the reference compound ascorbic acid (6.97 ± 0.01 mmol TE/g) [17].

Fruiting body extracts of *Pleurotus sajor-caju (Fr.) Singer* exhibited some extent of antistaphylococcal activity against MSSA and MRSA with a MIC value of 10 mg/mL with the presence of compounds such as *p*-hydroxybenzoic acid (**7**), *p*-Coumaric acid (**8**) and cinnamic acid (**9**) [72,73]. Cytotoxicity assay of the fungal fruiting body extract demonstrated strong inhibition against growth of non-small cell lung carcinoma (NCI-H460), breast (MCF-7) and cervical (HeLa) carcinoma cells. *Pleurotus sajor-caju* extract does not display cytotoxicity against non-tumour cells (primary PLP2) even at a test concentration of 400 μg/mL [72]. Ethanol extract of mycelia of the oyster mushroom, *P. ostreatus,* demonstrated a broad-spectrum antibacterial activity. Activity against the Gram-positive *S. aureus* showed a 24 mm diameter of inhibition zone in paper disc assay [74]. In another study, ethyl acetate mycelial extract of *P. ostreatus* exhibited potent antistaphylococcal activity with an inhibition zone of 8.5 mm diameter against *S. pyrogens*, comparable to the activity of control drug kanamycin (12.5 mg/mL) [75]. *Pleurotus aureovillosus* fruiting body extract showed antistaphyloccocal activity against *S. aureus* by displaying 8 mm diameter of inhibition zone [76]. This fungus has high potential to produce antistaphylococcal active compounds for drug development with further investigations.

The tropical basidiomycete fungus *Laxitextum incrustatum* from Kenya produced anticancer and antimicrobial compounds active against *S. aureus* strains. The isolated compound, laxitextine A (**10**), was discovered to show the highest activity against *S. aureus* (DSM 346) and MRSA with MIC value of 7.8 μg/mL (17.9 μM). Comparable cytotoxicity effect was also observed for this cyathane xyloside derived compound with IC_50_ values of 2.0–2.3 μM against breast cancer cell line (MCF-7) [77].

*Caloboletus radicans* possessed fruiting bodies that tasted bitter and are inedible due to the content of calopin compounds. The compound 8-deacetylcyclocalopin (**11**) showed inhibition against *S. aureus* ATCC 25923 and SA-1199B (MIC 16 µg/mL), and against *S. aureus* XU212 and EMRSA-15 at a MIC value of 32 µg/mL. The compound showed no activity against *S. aureus* RN4220. Different derivatives of calopins (100 μM) were inactive against the prostate cancer cell line PC3 and liver hepatoblastoma cell line HepG2 [78].

The compounds isolated from fruiting bodies of *Stereum hirsutum* mushroom were tested for different bioactivities. The isolated compounds, benzoate derivatives 1 (**12**) and 2 (**13**) revealed a strong antistaphylococcal activity with MIC value of 25 μg/mL and a significant cytotoxicity effect. However, derivative 1 (**12**) displayed stronger cytotoxicity against the growth of A549 cell line with an IC_50_ value of 13.14 ± 0.89 μM. Hence, this secondary metabolite was identified as a potential therapeutic agent [79].

Mycelia culture filtrate extract of *Pyrofomes demidoffi* exhibited strong inhibition against the in vitro growth of Gram-positive bacteria. *Staphylococcus* spp. were the most susceptible among the tested Gram-positive bacteria. Mycelial culture filtrate extract impregnated onto discs produced an inhibition zone with diameter of 46–47 mm against *S. aureus* strains. This indicated that culture filtrate extract of the fungus has a wide spectrum of activity against an array of bacteria strains [80].

The mushroom *Agaricus bisporus* identified from Njoro Kenya was tested for antimicrobial activity against an array of test bacteria and fungi. The mushroom metabolites from methanol extract revealed weak activity against Gram-positive bacteria. This was supported by diameters of inhibition zone of 16 ± 0.1 mm and 11.75 ± 0.96 mm against *S. aureus* exhibited by the mushroom extract from two different studies [81,82]. The metabolites responsible for the antistaphylococcal activities are 2,4-dihydroxybenzoic (**14**) and protocatechuic acid (**15**) [81]. Fruiting body extract of *A. brunnescens* prepared in chloroform and acetone solvents showed a higher antistaphylocccal activity with MIC value lower than 100 µg/mL [83].

Studies carried out by Reid et al. [84] presented that the fruiting body extracts of edible mushrooms from Zimbabwe, including *Amanita zambiana, Cantharellus miomboensis, Cantharellus symoensii, Cantharellus heinemannianus* and *Lactarius kabansus,* showed weak antistaphylococcal activity with inhibition zone range of 6.5–8.67 mm [84]. However, the non-edible mushroom *Trametes strumosa* had displayed slightly more potent activity than those edible mushrooms with a diameter of inhibition zone (DIZ) of 9.5 mm against *S. aureus*. Methanol extract of *Boletelus edulis* showed good activity against *S. aureus* with MIC value of 10.25 µg/mL [82].

The ethanol and aqueous extracts of *Auricularia sp*. showed activity against *S. aureus* with MIC value of 0.83 ± 0.29 µg/mL. High susceptibility of *S. aureus* towards the ethanol and aqueous extracts of *Termitomyces* sp. was observed with a MIC value of 0.67 ± 0.29 µg/mL. The extracts of this mushroom possessed potent antistaphyloccoccal activity [85]. On the other hand, the tested extracts of *Fomes fomentarius* showed lower antistaphyloccoccal activity with MIC value of 125 to 250 µg/mL when compared to the conventional antibiotic (ampicillin) with MIC value of 0.5 µg/mL [86].

Hot alkaline extracts obtained from fruiting bodies of the wild basidiomycete *Grifola frondosa* were examined for their antistaphyloccoccal activity. The antibacterial activity was determined through disc diffusion, Minimum Inhibitory Concentration (MIC) and Minimum Bactericidal Concentration (MBC) assays. The extract showed strong antistaphyloccoccal activity (Diameter of Inhibition Zone = 24 mm), almost similar to the control drug tetracycline (DIZ = 30 mm). This supported the strong MIC value observed for the fungal extract against *S. aureus* (39 µg/mL) [87].

Three wild mushrooms, *Trametes* sp. 1 (Arabuko-Sokoke forest), *Trametes* sp. 2 (Kakamega forest) and *Microsporus* sp. had shown promising antibacterial activity against *S. aureus* ATCC 25923). The chloroform, ethanol and hot water extracts of *Trametes* sp. 1 demonstrated good growth inhibitory activities against *Staphylococcus*. The MIC values (0.50–0.83 ± 0.29 mg/mL) against *S. aureus* indicated that the bacterium was highly susceptible to all of the tested extracts. Moreover, MRSA strain with MIC values of 0.83–1.00 mg/mL showed susceptibility towards the chloroform and ethanol extracts. MIC value of 0.50 mg/mL of *Trametes* sp. 2 displayed that *S. aureus* was the most susceptible to hot water extract. The chloroform and water extracts showed statistically significant antistaphylococcal activities compared to ethanol extract. The strong antistaphyloccoccal activity of *Microsporus* sp. was observed at MIC value of 0.67–1.00 mg/mL [88].

*Agaricus brunnescens* Peck and *Lactarius vellereus* (Fr.) Fr originated from Black Sea Region of Turkey possessed high antistaphylococcal activity in the chloroform and acetone extracts with a MIC value of 39 µg/mL. The author reported that a MIC value of less than 100 µg/mL was an indicator for high inhibitory activity [83].

Methanolic extracts of various mushrooms have shown a higher antibacterial activity against Gram-positive bacteria such as *S. aureus*. The basidiomycete *Lentinus* sp. presented potent antistaphyloccoccal activity with inhibition zone of 16 mm followed by antibacterial activity of *Schizophyllum commune* against *S. aureus* (9 mm) [76]. The extract and purified fraction of wild basidiomycete *Lentinus quercina* was tested against *S. aureus* and MRSA using agar well diffusion and disc diffusion assays. Extracts and the purified fraction of the macrofungus displayed a wide range of inhibition against *S. aureus* strains (8.7–22 mm) and MRSA strain (4–19 mm). The MIC results (3.125 to 6.25 µg/mL) showed that the wild basidiomycete has potent antistaphyloccoccal activity against both ATCC and clinical strains of *Staphylococcus* [89]. Oxalic acid from *L. edodes* (Berk.) Pegler was reported to be responsible for the growth inhibition of *S. aureus* [19].

Secondary metabolites from *Drechslera halodes* were investigated for potential antibacterial activity using disc diffusion and MIC assays. Fungal culture filtrate extracts displayed growth inhibition zone against *S. aureus* with a DIZ of 15 mm. The purified compound identified as 6-allyl-5,6-dihydro-5-hydroxypyran-2-one (**16**), which belongs to aromatic esterase group, revealed an MIC value of 25 µg/mL against *S. aureus*. The presence of other bioactive secondary metabolites in the culture filtrate extract of *D. halodes* attributed to a higher inhibitory activity against *S. aureus* [90].

*Lewia infectoris* derived bioactive compound namely pyrrocidine C (**17**) showed strong activity against *S. aureus* ATCC 29213 at MIC of 2 µg/mL. Previous reports mentioned that pyroccidine A and B are proven antibacterial against Gram-positive bacteria with similar MIC value of pyrrocidine C even though their relative stereochemistry is different to each other [91].

Fungus from genus *Cortinarius* produced compounds such as 6-methylxanthopurpurin-3-*O*-methyl ether (**18**), (1S,3S)-austrocortilutein (**20**), (1S,3R)-austrocortilutein (**21**), (1S,3S)-austrocortirubin (**22**) and erythroglaucin (**25**), which exhibited anti-*Staphylococcus* activity. The anthraquinones physcion (**19**) and emodin (**24**) exhibited the greatest potency against both Gram-positive and -negative bacteria. Ethanol fraction of most *Cortinus* sp., namely, *C. ardesiacus*, *C. archeri*, *C. austrosaginus*, *C. austrovenetus*, *C. austroviolaceus*, *C. coelopus*, *C. [Dermocybe canaria]*, *C. clelandii*, *C. [D. kula]*, *C. memoria-annae*, *C. persplendidus*, *C. sinapicolor* and *C. vinosipes* demonstrated IC_50_ values of ≤0.09 mg/mL against *S. aureus* [92].

*Lactarius piperatus*, *L. camphorates*, *L. volemus*, *Chanterellus cibarius*, *Ramaria flava*, *Macrolepiota procera*, *Leatiporus sulphureus*, *L. delicious*, *Boletus edulis*, *Hydnum repandum* and *Cortinarius* sp. are edible mushrooms commonly found in Black Sea Region of Turkey. The crude extracts of these wild mushrooms showed good activity against *S. aureus* with DIZ values ranging from 8.50 ± 1.00 to 11.75 ± 0.50 mm [82].

In another study carried out by Bala et al. [12], certain genera of macrofungi, namely, *Hohenbuehelia, Amanita* and *Agaricus* revealed strong antibacterial activity against *S. aureus* in their extracts. Within each sterile 96-well plate, the fungal fruiting body extract samples (ethanol and aqueous extracts) were prepared in serial dilutions. The initial absorbance reading was recorded as t_0_, and subsequently the t_22_ absorbance reading was taken after incubation with fungal extracts for 22 h at 37 °C. The growth inhibition percentage was measured with formula: % inhibition = {1 − (t_22_ − t_0_)/(C_22_ − C_0_)} × 100, where C_0_ is absorbance value of negative wells and C_22_ is the value corresponds to 22 h post incubation of negative wells. Both the water and ethanol extracts of *Hohenbuehelia* sp. exhibited strong inhibition against *S. aureus* at all four test concentrations (6.25%, 12.5%, 25% and 50%) as shown in Table S2.

## 4. Antibacterial Natural Products from Basidiomycetes against Other Drug-Resistant Pathogens

*Staphylococcus aureus*, *Enterococcus* spp., *Enterobacteriaceae*, *Pseudomonas aeruginosa* and *Acinetobacter* spp. have significant effects on contributing to the emergence of antimicrobial resistance that greatly impacts on healthcare system [93]. The presence of extended-spectrum beta-lactamase (ESBL) *Escherichia coli* strains causes high concern especially in healthcare setting due to their multidrug resistance properties. Some of the activities shown by secondary metabolites from Basidiomycetes against other drug-resistant pathogens (other than *S. aureus*) are given in Table S3.

*Quambalaria cyanescens* (Basidiomycota: Microstromatales) strains isolated from different insect species such as *Scolytus intricatus* (Bulgaria), *Phloetribus scarabeoides* (Croatia), *Scolytus amygdali* (Syria) and bark beetle feeding on *Arbutus unedo* (Tunnisia) were compared. Naphthoquinone derivatives, which are bright colored pigments identified from *Q. cyanescens*, exhibited potential antibacterial activity against *E. coli* with a DIZ value of 2–3mm. Quambalarine A (**26**) and mompain (**27**) are mainly active against bacterial strains. The antimicrobial activity described for both these compounds could be related to their selectivity towards mitochondria which centers the cellular energetic metabolism. Mompain (**27**) enables to transform the mitochondrial networks into vesicular formations while complete disappearance of mitochondria was observed when cells were treated with quambalarine B which leads to apoptosis phase. This indicated that alteration in mitochondrial function could be a potential mechanism of action for the compound in cells [11].

Ethanolic extract of *Pleurotus sajur-caju* displayed antimicrobial activity against *P. aeruginosa* and *Klebsiella pneumoniae* while the methanolic extract was active against *E. coli* [94,95]. *Pleurotus sajor-caju* ethanolic extract is composed of lipophilic compounds, *p*-Hydroxybenzoic (**7**), *p*-coumaric (**8**) and cinnamic acids (**9**) at respective concentration: 66 ± 4, 43.7 ± 0.9 and 37.5 ± 0.3 μg/g extract [72]. A previous report mentioned that the most suitable solvent to extract maximum antimicrobial bioactive compounds from the fungus is ethanol [94].

High anti-Gram-positive bacteria activity was observed in extracts of *Fistulina hepatica* and *Tapinella atrotomentosa* with MIC values ranging between 12.5–100 µg/mL. However, *F. hepatica* extract was inactive, whereas *T. atromentosa* was highly active against Gram-negative bacteria. The justification behind the observed difference could be that of type of solvent used for extraction process which fractioned out different compounds, and time and place of collection of the sample which may have affected the activity as well [17].

*Tapinella atrotomentosa* extracted by chloroform exhibited a broad spectrum of antibacterial activity against Gram-positive bacteria, ESBL *E. coli* and multidrug-resistant *P. aeruginosa* and *Acinetobacter baumanii*. Osmundalactone (**4**), 5-hydroxy-hex-2-en-4-olide (**5**), spiromentin C (**6**) and spiromentin B are metabolites with a terphenylquinone skeleton isolated from this fungus and assessed for their antibacterial activity using the broth microdilution assay. Although osmundalactone (**4**) and spiromentin C (**6**) were highly active against *A. baumanii* and *E. coli*, 5-hydroxy-hex-2-en-4-olide (**5**) was the most active antibacterial constituent against these multidrug-resistant bacteria [64]. Secondary metabolites from *Agaricus bisporus* and *Trametes gibbosa* inhibited growth of Gram-negative bacteria in previous studies (Table S3). Inhibition of the bacterial growth by *T. gibbosa* extracts was significantly higher when compared to inhibition exhibited by *A. bisporus* extracts [17].

Crude extracts from fruiting bodies and biomass of *Ganoderma* were reported to exhibit in vitro antibacterial effect against *Staphylococcus* spp., *E. coli* and *Enterobacter* spp. [96]. However, variations are present among *G. lucidum* strains regarding yield and production of bioactive compounds and their antimicrobial activity [69]. The methanolic extracts of biomass and fruiting body of *G. lucidum* strains were found to show low antibacterial activity against *K. pneumoniae* and *E. coli* while the aqueous extracts of biomass and fruiting body exhibited minimum inhibition of growth against *Pseudomonas* spp. Generally, methanolic extracts showed effective inhibition zone as compared to aqueous extracts [15].

Chloroform extract of *Auricularia* spp. exhibited activities with significant difference against resistant pathogens such as *E. coli*, *K. pneumoniae* (ATCC 13883) and *P. aeruginosa*. Ethanol and aqueous extracts displayed limited activity against *E. coli* and *P. aeruginosa*. From the results, extracts of *Auricularia* spp. were found to be less active against the Gram-negative bacteria *E. coli* [82]. Both chloroform and ethanol extracts of *Auricularia* and *Termitomyces* spp. exhibited a weaker antimicrobial activity. This can be explained by the absence of bioactive compounds in the extracts or the loss of functionality due to deviations associated with difference in the amount and type of bioactive constituents present in the extracts [82]. The dichloromethane, methanol, aqueous and cyclohexane extracts of *Fomes fomentarius* showed potential activity against *E. coli* at MIC 125 µg/mL. Generally, high total polyphenol content was found in methanol and aqueous extracts that possessed strong antimicrobial activity [93].

*Drechslera halodes* produced a potential bioactive compound that exhibited growth inhibition activity against *E. coli* with a DIZ value of 12 mm diameter and MIC 50 µg/mL. Secondary metabolites excreted into the culture medium during submerged fermentation attributed to this potential antibacterial activity. There are no cytotoxicity effects observed for the bioactive compound isolated from the fungal culture filtrate. The responsible alkaloid compound was identified as 6-allyl-5,6-dihydro-5-hydroxypyran-2-one (**16**). The mode of action of this compound was related to its inhibition effects on DNA synthesis [90].

Bioactive compounds isolated from fruiting bodies of *Cortinarius* sp. were investigated for anti-*Staphylococcus* and anti-*Pseudomonas* activity using microdilution assay. Compound (**19**) was more active against *P. aeruginosa* than *S. aureus*. Compounds (**20**) to (**24**), however, displayed lower activity against *P. aeruginosa*. *Cortinarius abnormis*, *C. austroalbidus*, *C. [D. kula]* and *C. persplendidus*, and eleven unknown *Cortinarius* spp. were reported to exhibit anti-*Pseudomonas* activity with IC_50_ ≤ 0.09 mg/mL [92].

Potential antibacterial compounds have been isolated from organic extracts of the fungus *Pleurotus eous*. The petroleum ether extracts of *P. eous* possessed several fatty acids compounds such as stearic acid (**28**), heptadecanoic acid (**29**) and tartronic acid (**30**) that attributed to high growth inhibitory activity against *E. coli* (DIZ = 11 ± 0 mm) than *K. pneumoniae.* The antimicrobial activity of this fungus is related to the presence of fatty acids which traditionally could be used for pain, fever and inflammatory disorders [97].

## 5. Conclusions

The present review focuses on antistaphyloccocal activity of basidiomycetes globally and their isolated secondary metabolites. An exhaustive literature search was performed and only those basidiomycete extracts (and isolated compounds) with positive antistaphyloccoccal activity were included. For further extensive scientific studies, it will certainly prove useful to investigate the metabolites derived from this fungal group.

The emergence of several staphylococcal resistance strains linked to nosocomial infections requires a new antimicrobial solution. Literature reports indicated that extracts and/or compounds from various basidiomycete species exhibited potential antibacterial activity against *Staphylococcus* strains. Increasing reports in recent years have also shown the potential ability of some natural extracts in potentiating the activity of standard antibiotics [88,90]. Further pharmacological studies to look at synergistic interactions between antibiotics and these natural products can greatly improve the potential activities of moderately toxic antistaphylococcal compounds.

The numerous methodologies used for the assessment of antimicrobial activity of basidiomycete extracts or isolated compounds made it difficult to compare between the published data. The factors involved like type of solvent, conditions such as temperature and time, and the structure of compounds that causes variation in the extraction of bioactive compounds has to be optimised to attain a greater overall output and efficiency of the target compounds. The geographic location, different fungal cultivation method and types of growth media could possibly affect the content and amount of active compounds in the extracts [90]. Thus, the standardisation of methods and establishment of cut-off values for activity is important. The information on mechanisms of action of different fungal-derived compounds could assist in the development of new active pharmaceutical ingredients with interesting or novel activity. Moreover, the use of cytotoxicity assays is also important for the evaluation of effects of a compound in human at the in vitro efficacy concentrations studied.

The available literature studies are mostly focused on screening of antibacterial properties of basidiomycete extracts. The prospect of discovering new secondary metabolites from them is highly valued. The lack of data on mechanism of action of the antimicrobial compounds from Basidiomycete fungus does not support their use as a sole antibiotic. Further studies are needed before these compounds are made available for human use. Identifying individual compounds that exhibit antibacterial activity and explaining their mode of action is inevitable in drug discovery. This provides an avenue for the creation of pharmaceuticals that are effective against selected microorganisms resistant to traditional therapy in tandem with the growing multidrug resistance crisis in recent decades.

## Supplementary Materials

The following are available online, Table S1: Antistaphylococcal activity reported from Basidiomycete fungi; Table S2: Antistaphylococcal activity showed by fruiting bodies of Basidiomycetes in water and ethanol extracts at various concentration; Table S3: Antibacterial activity of Basidiomycete compounds against other pathogens; Figure S1: Structures of antimicrobial compounds isolated from Basidiomycetes.
