# Supplementary material for: A Review on Antistaphylococcal Secondary Metabolites from Basidiomycetes
Source: Molecules. 2020 Dec 11;25(24):5848. doi: 10.3390/molecules25245848 (PMC7764641; doi:10.3390/molecules25245848)
Supplement: Supplementary file 1 [file molecules-25-05848-s001.zip › supplementary/molecules-967866 figures.pdf]

Supplementary Material 2.0

Figure 1: Structures of antimicrobial compounds isolated from Basidiomycetes.

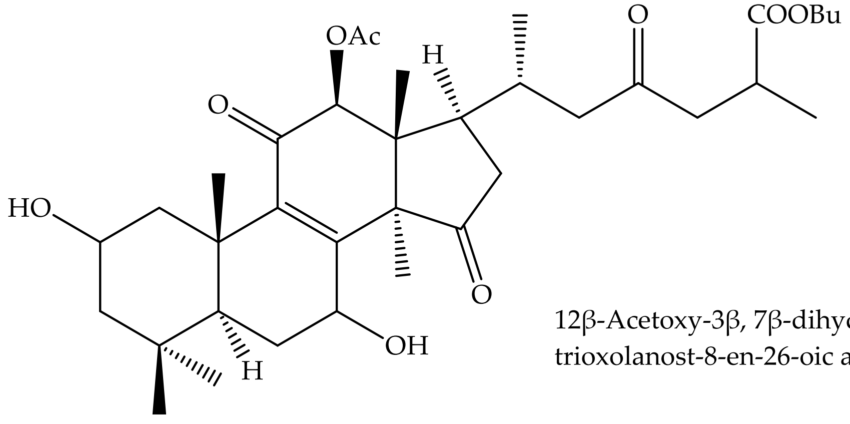

12β-Acetoxy-3β, 7β-dihydroxy-11, 15, 23-trioxolanost-8-en-26-oic acid butyl ester **(1)**

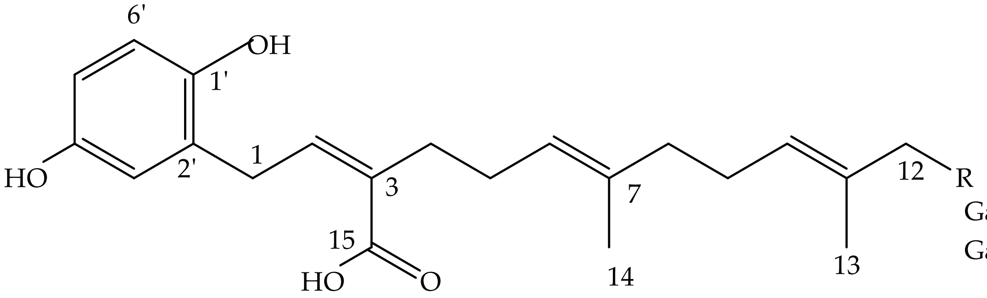

Ganomycin A: R=OH **(2)**
Ganomycin B: R=H **(3)**

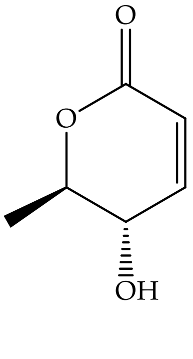

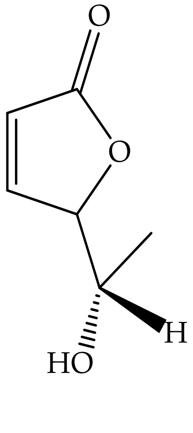

Osmundalactone **(4)**

5-Hydroxy-hex-2-en-4-olide **(5)**

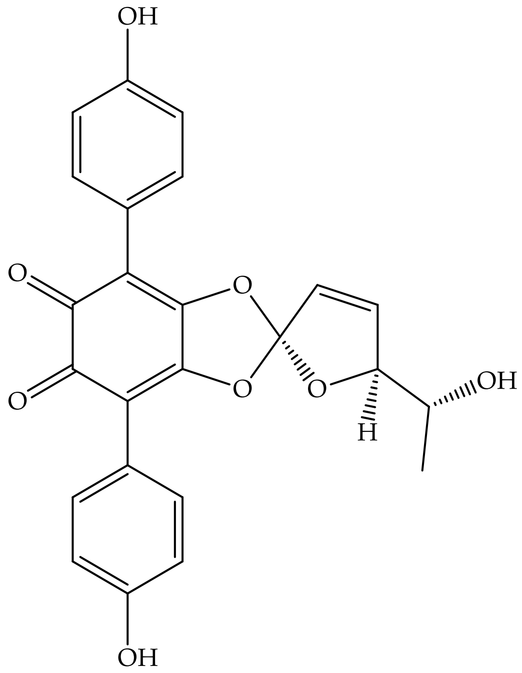

Spiromentin C **(6)**

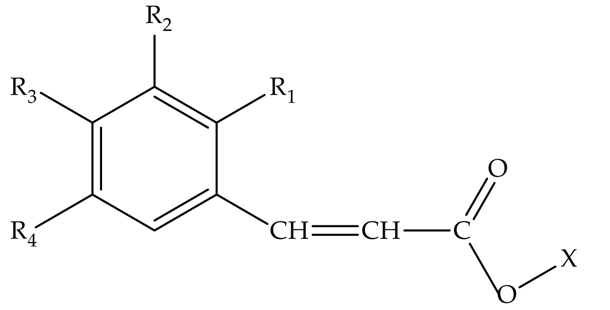

p-Coumaric acid: X=CHCHCOOH; R_1=H; R_2=H; R_3=OH; R_4=H **(8)**

Cinnamic acid: X=CHCHCOOH; R_1=H; R_2=H; R_3=H; R_4=H **(9)**

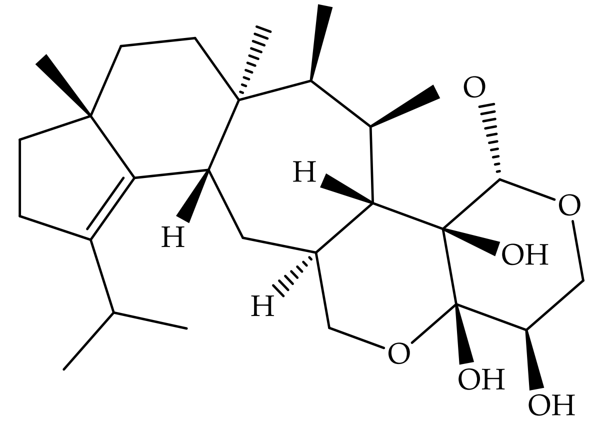

Laxitextine A **(10)**

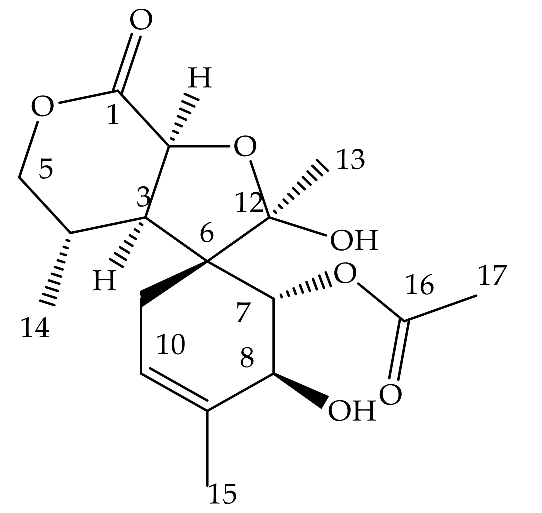

8-Deacetylcyclocalopin B **(11)**

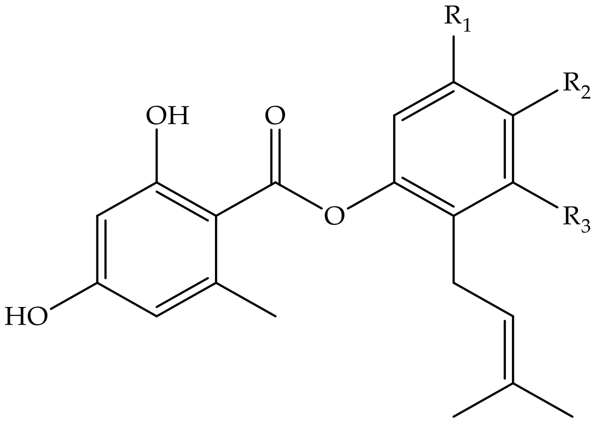

Benzoate derivative 1: R_1=H; R_2=OH; R_3=OCH_3 **(12)**
Benzoate derivative 2: R_1=H; R_2=OH; R_3=H **(13)**

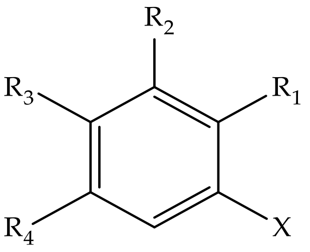

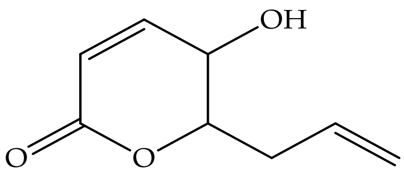

p-Hydroxybenzoic acid: X=COOH; R_1=H; R_2=H; R_3=OH; R_4=H **(7)**
2,4-Dihydroxybenzoic acid: X=COOH; R_1=OH; R_2=H; R_3=OH; R_4=H **(14)**
Protocatechuic acid: X=COOH; R_1=H; R_2=H; R_3=OH; R_4=OH **(15)**

6-allyl-5,6-dihydro-5
hydroxypyran-2-one **(16)**

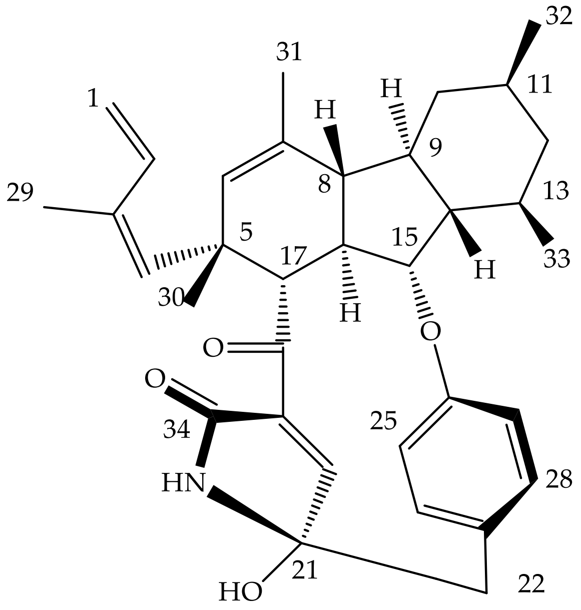

Pyrrocidine C **(17)**

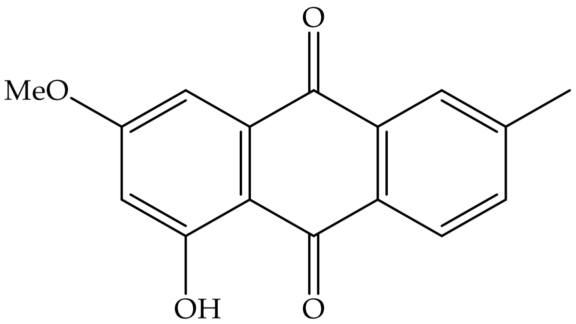

6-methylxanthopurpurin-3-*O*-methyl ether (**18**)

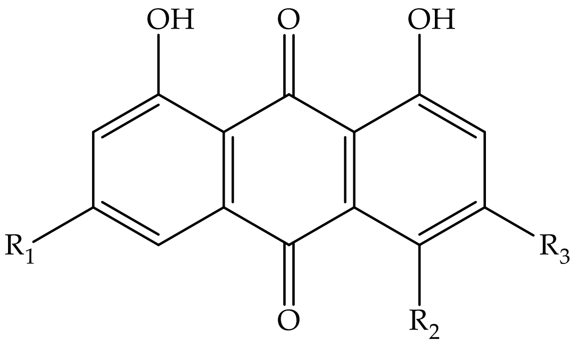

Physcion: R_1=Me; R_2=H; R_3=OMe (**19**)
Emodin: R_1=Me; R_2=H; R_3=OMe (**24**)
Erythroglaucin: R_1=Me; R_2=H; R_3=OMe (**25**)

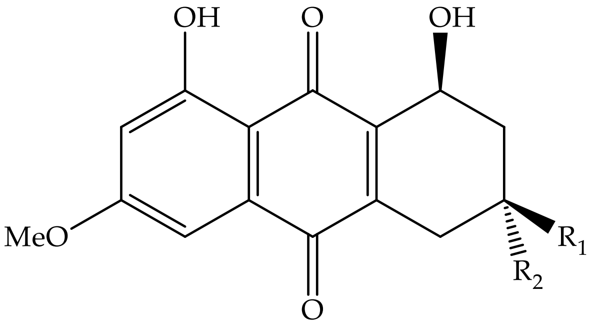

(IS,3S) Austrocortilutein: R_1=OH; R_2=Me (**20)**
(IS,3R) Austrocortilutein: R_1=Me; R_2= OH (**21**)

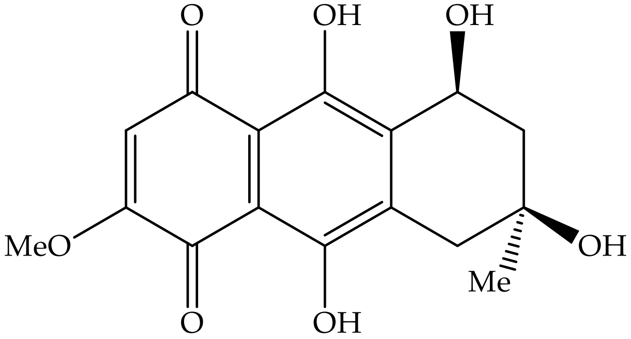

Austrocortirubin (**22**)

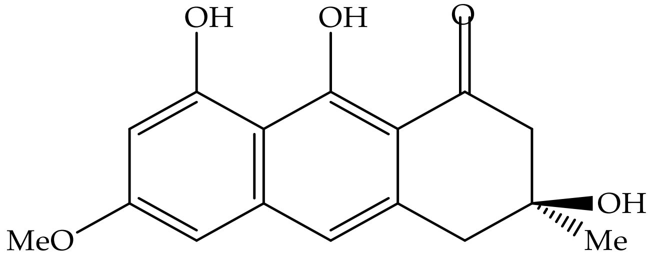

Torosachrysone (**23**)

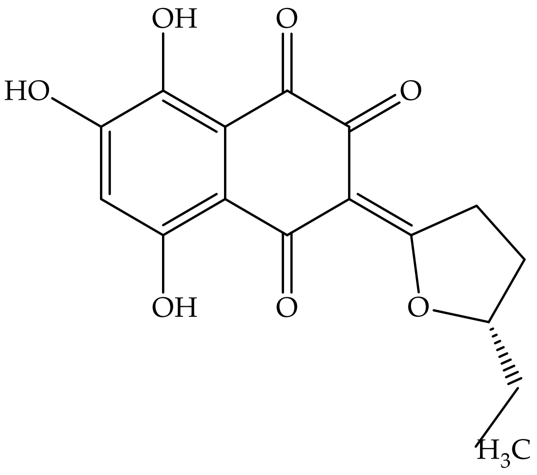

Quambalarine A **(26)**

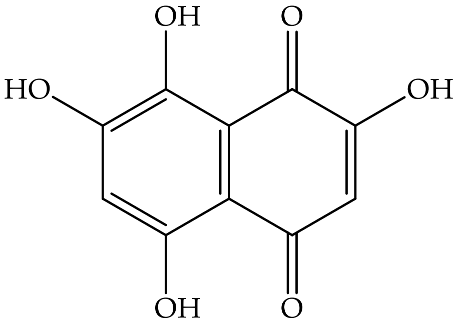

Mompain **(27)**

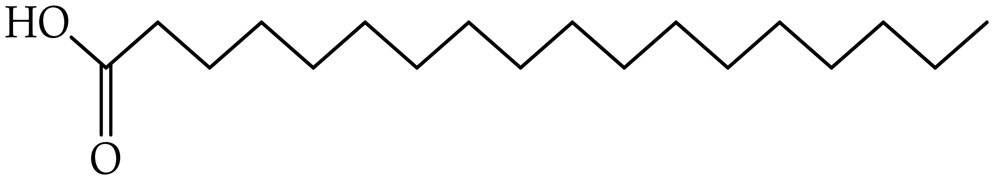

Stearic acid **(28)**

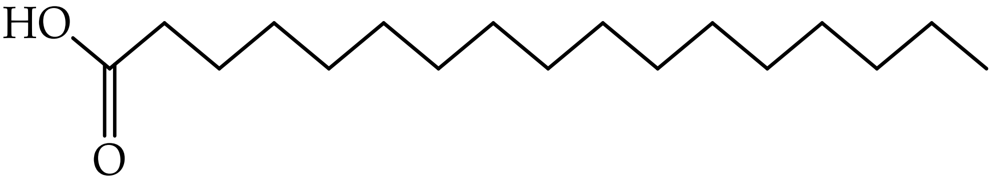

Heptadecanoic acid **(29)**

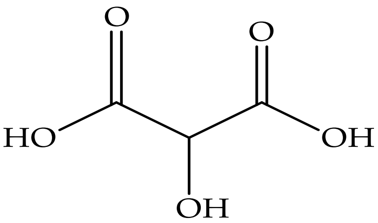

Tartronic acid **(30)**
