# Supplementary material for: A Review on Antistaphylococcal Secondary Metabolites from Basidiomycetes
Source: Molecules. 2020 Dec 11;25(24):5848. doi: 10.3390/molecules25245848 (PMC7764641; doi:10.3390/molecules25245848)
Supplement: Supplementary file 1 [file molecules-25-05848-s001.zip › supplementary/molecules-967866 supp.docx]

**Supplementary Material 1.0**

Table 1: Antistaphylococcal activity reported from Basidiomycete fungi.

| Species | Origin | Natural Product | | Antistaphyloccal Activities | | | Mode of action | Reference |
| --- | --- | --- | --- | --- | --- | --- | --- | --- |
|  |  | Compounds Isolated (F/M) | Solvent Extract (F/M) | DIZ  (mm) | MIC  (ug/ml) | MBC  (ug/ml) |  |  |
| *Ganoderma lucidum* | Europe | 12β-acetoxy-3β, 7β-dihydroxy-11, 15, 23-  trioxolanost-8-en-26-oic acid butyl ester(F) **(1)** | - | - | 68.5uM | - | NA | 70 |
| *Ganoderma pfeifferi* | Europe | Ganomycin A(F) **(2)**  Ganomycin B(F) **(3)** | - | 15-24  (*S.aureus* ATCC 6538, ATCC 25923, ATCC 29213, SG 511) | 25.0 | - | NA | 19;71 |
| *Tapinella atrotomentosa* | Szeged (Hungary) | Osmundalactone **(4)**  5-Hydroxy-hex-2-en-4-olide **(5)**  Spiromentin C **(6)** | Methanol(F) | - | 250  (MRSA SZMC 6270) | - | NA | 17  . |
| *Pleurotus sajor-caju (Fr.) Singer* | South of Brazil | *p*-Hydroxybenzoic acid **(7)**  *p*-Coumaric acid **(8)**  Cinnamic acid **(9)** (F) | Ethanol | - | 10000  (*S. aureus* & MRSA) | - | NA | 72 |
| *Pleurotus ostreatus* | Savar, Dhaka | - | Ethyl acetate(M) | 8.5 (*S. pyrogens*) | - | - | NA | 76 |
| *Pleurotus aureovillosus* | Kampo Chinese | - | Methanol(M) | 8.0  (*S. aureus*) | - | - | NA | 76 |
| *Laxitextum incrustatum* | West Kenya (Kakamega forest) | Laxitextine A(F) **(10)** | - | - | 7.8  (*S. aureus* & MRSA) | - | NA | 77 |
| *Caloboletus*  *radicans* | Bavaria, Germany | 8-Deacetylcyclocalopin B(F) **(11)** | - | - | 16.0  (*S. aureus* ATCC 25923 & SA-1199B)  32.0  (*S. aureus* XU212 & EMRSA-15) | - | NA | 78 |
| *Stereum hirsutum* | Tibetan Plateau | Benzoate derivate 1 **(12)**  Benzoate derivate 2 **(13)** (F) | - | - | 25.0  (*S. aureus* & MRSA) | - | NA | 79 |
| *Pyrofomes demidoffi* | South West of Addis Ababa (Ethopia-Menagesha forest) | - | Ethyl acetate (M) | 46-47  (*S. aureus)* | - | - | NA | 81;82 |
| *Agaricus bisporus* | Southeast of Nairobi | 2,4-dihydroxybenzoic **(14)**  Protocatechuic acid **(15)** | Methanol(F) | 16 ± 0.1  11.75 ± 0.96  (*S. aureus*) | *-* | - | NA | 83 |
| *Amanita zambiana* | Zimbabwe | - | Methanol(F)  Ethanol(F) | 7.0 ± 0.0  7.33 ± 0.58 | - | - | NA | 82 |
| *Cantharellus miomboensia* | Zimbabwe | - | Methanol(F)  Ethanol(F)  Acetone (F) | 6.5 ± 0.00  8.16 ± 0.29  7.67 ± 0.29 | - | - | NA | 82 |
| *Cantharelllus symoensii* | Zimbabwe | - | Methanol(F)  Ethanol (F)  Acetone(F) | 7.23-± 0.25  8.2 ± 0.76  8.67 ± 0.58 | - | - | NA | 82 |
| *Cantharellus heinemannianus* | Zimbabwe | - | Ethanol(F)  Acetone(F) | 8.83 ± 0.29  8.07 ± 0.12 | - | - | NA | 82 |
| *Lactarius kabansus* | Zimbabwe | - | Ethanol(F) | 7.83 | - | - | NA | 82 |
| *Trametes strumosa* | Zimbabwe | - | Acetone(F) | 9.5 | - | - | NA | 82 |
| *Boletus edulis* | Zimbabwe  Black Sea Region of Turkey | - | Ethanol (F)  Acetone(F)  Methanol(F) | 7.50 ± 0.58  7.0 ± 0.0  10.25 ± 0.50 | - | - | NA | 82;83 |
| *Auricularia sp.* | Kakamega National Reserve Forest in Kenya | - | Chloroform  Ethanol  Aqueous | - | 1.00 ± 0.00  (*S. aureus)*  1.00 ± 0.00 (MRSA)  0.83 ± 0.29  *(S. aureus)*  1.00 ± 0.00 (MRSA)  0.83 ± 0.29  *(S. aureus)*  1.00 ± 0.00  (MRSA) | 1.00 ± 0.00  1.33 ± 0.58  0.83 ± 0.29  1.00 ± 0.00  0.67 ± 0.29  1.00 ± 0.00 | NA | 86 |
| *Termitomyces sp.* | Kakamega National Reserve Forest in Kenya | - | Chloroform  Ethanol  Aqueous | - | 0.83 ± 0.29  *(S. aureus)*  1.33 ± 0.58 (MRSA)  0.67 ± 0.29  *(S. aureus)*  0.83 ± 0.29 (MRSA)  0.67 ± 0.29  *(S. aureus)*  0.83 ± 0.29 (MRSA) | 0.83 ± 0.29  1.00 ± 0.00  0.67 ± 0.29  1.00 ± 0.00  0.50 ± 0.00  0.83 ± 0.29 | NA | 86 |
| *Agaricus brunnescens Peck* | Bozyazi (Mediter-ranean Sea) | - | Acetone & chloroform(F)  Methanol(F) | - | 39 *(S. aureus)*  156  *(S. aureus)* | - | NA | 83 |
| *Lactarius vellereus* | Bozyazi (Mediter-ranean Sea) | - | Acetone & chloroform(F) | - | 39  *(S. aureus)* | - | NA | 83 |
| *Fomes fomentarius* | Mountain Avala (Serbia) | - | Cyclohexane(F)  Dichloromethane, methanol & aqueous(F) | - | 250  *(S. aureus)*  125  *(S. aureus)* | - |  | 86 |
| *Grifola frondosa* | Bojcinska forest (Republic of Serbia) |  | Hot alkali(F) | 19.0 - 24.0  *(S. aureus)* | 39  *(S. aureus)* | 2500 | NA | 87 |
| *Trametes spp.* | Arabuko-Sokoke and Kakamega National Reserve forests in  Kenya. | - | Chloroform  Ethanol  Aqueous |  | 0.83 ± 0.29  *(S. aureus* ATCC 25923*)*  1.17 ± 0.76  (MRSA ATCC 33591)  0.67 ± 0.29  *(S. aureus)*  0.83 ± 0.29 (MRSA)  0.67 ±0.29  *(S. aureus)*  0.83 ± 0.29  (MRSA) | 0.83 ± 0.29  1.00 ± 0.0  0.83 ± 0.29  1.00 ± 0.00  0.67 ± 0.29  1.00 ± 0.00 | NA | 88 |
| *Microsporus spp.* | Arabuko-Sokoke and Kakamega National Reserve forests in  Kenya. | - | Chloroform  Ethanol  Aqueous | - | 0.67 ± 0.29  *(S. aureus)*  0.83 ± 0.29  (MRSA)  0.67 ± 0.29  *(S. aureus)*  0.83 ± 0.29  (MRSA)  0.50 ± 0.00  *(S. aureus)*  0.83 ± 0.29  (MRSA) | 1.00 ± 0.0  1.00 ± 0.0  0.83 ± 0.29  0.83 ± 0.29  0.50 ± 0.00  0.83 ± 0.29 | NA | 88 |
| *Lentinus sp.* | Kampo Chinese | - | Methanol | 16.0  *(S. aureus)* | - | - | NA | 76 |
| *Sxhizophyllum commune* | Kampo Chinese | - | Methanol | 9.0  *(S. aureus)* | - | - | NA | 76 |
| *Pleurotus aurevillosus* | Kampo Chinese | - | Methanol | 8.0  *(S. aureus)* | - | - | NA | 76 |
| *Lentinus quercina* | Nigeria | - | Raw & Fermented Extract:  RET  FET  Purified fraction:  RPE  RMT | 9-10.7  *(S. aureus)*  8-18  (MRSA)  -  11.3-13.0  (MRSA)  18-22  *(S. aureus)*  10.2 (MRSA)  14-16  *(S. aureus)*  13.2-19.0  (MRSA) | 3.125-6.25  *(S. aureus)*  6.25-25.0  (MRSA)  3.125  (*S. aureus*)  6.25  (MRSA)  -  -  -  - | 12.5  12.5-25.0  25.0  12.5  -  -  -  - | NA | 76;89 |
| *Lactarius piperatus* | Black Sea Region of Turkey | - | Methanol | 10.75 ± 0.96  *(S. aureus)* | - | - | NA | 82 |
| *Lactarius camphorates* | Black Sea Region of Turkey | - | Methanol | 11.25 ± 0.96  *(S. aureus)* | - | - | NA | 82 |
| *Lactarius volemus* | Black Sea Region of Turkey | - | Methanol | 10.00 ± 0.50  *(S. aureus)* | - | - | NA | 82 |
| *Chanterellus cibarius* | Black Sea Region of Turkey | - | Methanol | 11.25 ± 0.50  *(S. aureus)* | - | - | NA | 82 |
| *Ramaria flava* | Black Sea Region of Turkey | - | Methanol | 9.50 ± 0.56  *(S. aureus)* | - | - | NA | 82 |
| *Macrolepoita procera* | Black Sea Region of Turkey | - | Methanol | 10.00 ± 0.82  *(S. aureus)* | - | - | NA | 82 |
| *Leatiporus sulphureus* | Black Sea Region of Turkey | - | Methanol | 11.00 ± 0.82  *(S. aureus)* | - | - | NA | 82 |
| *Lactarius delicious* | Black Sea Region of Turkey | - | Methanol | 8.50 ± 1.00  *(S. aureus)* | - | - | NA | 82 |
| *Hydnum repandum* | Black Sea Region of Turkey | - | Methanol | 11.75 ± 0.50  *(S. aureus)* | - | - | NA | 82 |
| *Drechslera halodes* | Basrah, Iraq | 6-allyl-5,6-dihydro-5-hydroxypyran-2-one (Culture filtrate)(**16**) |  | 30.0(*S.aureus)* | 25.00 | - | Inhibit synthesis of DNA | 90 |
| *Lewia infectoris*  SNB-GTC2402 |  | Pyrrocidine C(**17**) | - | - | 2.0 (*S.aureus* ATCC 29213) | - | NA | 91 |
| *Cortinarius sp* | Black Sea Region of Turkey | 6-methylxanthopurpurin-3-O-methyl ether(**18**)  Physcion(**19**)  (1S,3S)-Austrocortilutein(**20**)  (1S,3R)-Austrocortilutein(**21**)  (1S,3S)-Austrocortirubin(**22**)  Torosachrysone(**23**)  Emodin(**24**)  Erythroglaucin(**25**) | - | - | IC_50_ : ≥50  IC_50_ : 23  IC_50_ : 8  IC_50_ : 12  IC_50_ : 3  IC_50_ : 10  IC_50_ : 0.7  IC_50_ : ≥50 | - | NA | 92 |

Note: F=Fruiting body; M=Mycelium; DIZ=Diameter of Inhibition zone; MIC= Minimum Inhibitory Concentration; MIC_50_=Minimum Inhibitory Concentration required to inhibit 50% growth of organisms ;MIC_90_ = Minimum Inhibitory Concentration required to inhibit 90% growth of organisms; MBC= Minimum Bactericidal Concentration; Sample conc= Sample concentration; RET: raw *Lenzites quercina* extracted with ethanol; FET: fermented *Lenzites quercina* extracted with ethanol; RPE: pet-ether fraction; RMT: methanol fraction; NA: No data available; IC_50_ : 50% inhibitory concentration.

Table 2: Antistaphylococcal activity showed by fruiting bodies of Basidiomycetes in water and ethanol extracts at various concentration using a high-throughput 96 microplates bioassay procedure to determine the percentage of growth inhibition of *S.aureus*.

| Species | Origin | Solvent Extract | Sample concentration (%) | Growth inhibition (%) of *S. aureus* | References |
| --- | --- | --- | --- | --- | --- |
| *Agaricus aff.xanthodermus* | Queensland, Australia | Water  Ethanol | 12.5-50  6.25  50 | 50-75%  75-100%  50-75% | 12 |
| *Agaricus* sp. | Queensland, Australia | Water  Ethanol | 6.25-50.0  50 | 75-100%  75-100% | 12 |
| *Amanita* sp | Queensland, Australia | Water  Ethanol | 25-50  25-50 | 75-100%  75-100% | 12 |
| *Amanita flavella* | Queensland, Australia | Ethanol | 50  25 | 75-100%  50-75% | 12 |
| *Amanita ochrophylla* | Queensland, Australia | Water  Ethanol | 12.5-50  50 | 75-100%  50-75% | 12 |
| *Armillaria mellea* | Queensland, Australia | Water | 12.5-50 | 75-100% | 12 |
| *Calvatia* sp. | Queensland, Australia | Ethanol | 25-50  12.5 | 75-100%  50-75% | 12 |
| *Chlorophyllum molybdites* | Queensland, Australia | Water | 50 | 75-100% | 12 |
| *Cortinarius* sp. | Queensland, Australia | Water  Ethanol | 12.5-50  25-50 | 25-50%  75-100% | 12 |
| *Coprinus comatus* | Queensland, Australia | Water  Ethanol | 25-50  50 | 25-50%  75-100% | 12 |
| *Hohenbuehelia* sp. | Queensland, Australia | Water & Ethanol | 6.25-50 | 75-100% | 12 |
| *Psathyrella* sp. | Queensland, Australia | Water  Ethanol | 25-50  12.5.-50  6.25 | 50-75%  75-100%  50-75% | 12 |
| *Boletus* subsect. *luridi* | Queensland, Australia | Water  Ethanol | 50  25-50  12.5 | 75-100%  75-100%  50-75% | 12 |
| *Strobilomyces* sp. | Queensland, Australia | Water  Ethanol | 25-50  12.5 | 75-100%  50-75% | 12 |
| *Tylopilus* sp.1 | Queensland, Australia | Water  Ethanol | 6.25-50  50 | 75-100%  75-100% | 12 |
| *Tylopilus* sp.1 | Queensland, Australia | Ethanol | 25-50 | 75-100% | 12 |
| *Phallus multicolor* | Queensland, Australia | Water  Ethanol | 12.5-50  50 | 50-75%  75-100% | 12 |
| *Ramaria* sp.1 | Queensland, Australia | Water  Ethanol | 6.25-50  6.25 | 75-100%  50-75% | 12 |
| *Ramaria zippellii* | Queensland, Australia | Water  Ethanol | 25  50 | 50-75%  75-100% | 12 |
| *Ganoderma* sp | Queensland, Australia | Water  Ethanol | 50  25-50 | 75-100%  75-100% | 12 |
| *Lentinus* sp.3 | Queensland, Australia | Water  Ethanol | 12.5-50  25-50 | 75-100%  75-100% | 12 |
| *Fomitopsis lilacinogilva* | Queensland, Australia | Water  Ethanol | 25-50  6.25-50 | 75-100%  75-100% | 12 |
| *Craterellus* sp. | Queensland, Australia | Water | 50  12.5-25 | 75-100%  50-75% | 12 |
| *Cantharellus* sp. | Queensland, Australia | Water  Ethanol | 25-50  6.25-50 | 75-100%  75-100% | 12 |
| *Russula erumpens* | Queensland, Australia | Water | 50 | 75-100% | 12 |

Table 3: Antibacterial activity of Basidiomycete compounds against other pathogens.

| Species | Solvent Extract /Compound | *In-vitro* assay | | | Test Organisms | Mode of action | Reference |
| --- | --- | --- | --- | --- | --- | --- | --- |
|  |  | DIZ  (mm) | MIC  (ug/ml) | MBC (ug/ml) |  |  |  |
| *Quambalaria cyanescens* CCM 8372 | Quambalarine A **(26)**  Mompain **(27)** | 2-3  3 | - | - | *Escherichia coli* | Alter functionality of mitochondria | 11 |
| Tapinella atromentosa | Osmundalactone(**4**)  5-Hydroxy-hex-2-en-4-olide(**5**)  Spiromentin C(**6**) | - | 10/10  10/6  10/20 | - | ESBL *E. coli*/MACI  ESBL *E. coli*/MACI  ESBL *E.coli*/MACI | NA | 17 |
| *Pleurotus sajor-caju* (Fr.) Singer | p-Hydroxybenzoic(**7**)  p-Coumaric (**8**)  Cinnamic acids(**9**) | - | >20,000 | - | ESBL *E. coli*, *E. coli*, *Klebsiella pneumoniae*, ESBL *K. pneumoniae*, *Enterococcus faecalis*, *Pseudomonas aeruginosa* | NA | 72;94;95 |
| *Agaricus bisporus* | 2,4-dihydroxybenzoic (**14**)  Protocatechuic acid(**15**) | 9±0.2 to 15±0.3  10±0.2 to 22±0.2  10±0.1 to 20±0.2 | - | - | *E. fecalis*  *E. coli*  *P. aeruginosa* | NA | 17 |
| *Trametes gibbosa* | Chloroform | 14±0.1 to 23±0.2  17±0.2 to 24±0.1 | - | - | *E. coli*  *P. aeruginosa* | NA | 17 |
| *Auricularia* sp. | Ethanol | - | 2.00±0.0  1.33±0.58  2.00±0.0  1.67 ±0.58 | 2.00±0.0  1.33±0.58  2.00±0.0  1.67 ±0.58 | *E. coli* (clinical isolate)  *K.pneumoniae* ATCC 13883  *P. aeruginosa* (Clinical isolate)  *P. aeruginosa* ATCC 27853 | NA | 82 |
| *Termitomyces* sp. | Hot water | - | 0.83±0.29  0.83±0.29  1.00±0.0  0.83±0.29 | 1.67±0.58  0.83±0.29  0.83±0.29  0.83±0.29 | *E. coli* (clinical isolate)  *K. pneumoniae* ATCC 13883  *P. aeruginosa* (Clinical isolate)  *P. aeruginosa* ATCC 27853 | NA | 82 |
| *Fomes fomentarius* | Aqueous | 125 | - | - | *K. pneumoniae* NCIMB 9111, *E. coli* ATCC 25922, *Micrococcus luteus* ATCC 9341, *P. aeruginosa* ATCC 27853, *E. faecalis* ATCC 29212 | NA | 82 |
| *Grifola frondosa* | Hot alkali | 13.0 ± 0.0  6.7 ± 0.68 9.0±0.0 | - | - | *E. faecalis* ATCC 29212  *E. coli* ATCC 25922  *E. coli* (0157:H7) | NA | 87 |
| *Trametes* spp. | Chloroform  70% ethanol  Hot water | - | 1.33±0.58  1.00±0.00  1.00±0.00  1.33±0.58  1.00±0.00  0.83±0.29 | 1.67±0.58  1.33±0.58  1.33±0.58  1.00±0.00  1.00±0.00  0.83±0.29 | *E. coli* (clinical isolate), *K. pneumoniae* ATCC 13883, *P. aeruginosa* (Clinical isolate)  *P. aeruginosa* ATTC 27853  *P. aeruginosa* (Clinical isolate)  *E. coli* (clinical isolate), *K. pneumoniae* ATCC 13883, *P. aeruginosa* ATTC 27853  *P. aeruginosa* (Clinical isolate)  *E. coli* (clinical isolate), *K. pneumoniae* ATCC 13883, *P. aeruginosa* ATTC 27853 | NA | 17 |
| *Microsporus* spp. | Chloroform  70% ethanol/hot water | - | 2.00±0.00  1.33±0.58  1.67±0.58  1.67±0.58  1.00±0.00  1.33±0.58 | 2.00±0.00  1.67±0.58  1.33±0.58  1.67±0.58  1.33±0.58  1.00±0.00 | *E. coli* (clinical isolate)  *P. aeruginosa* (Clinical isolate)  *K. pneumoniae* ATCC 13883  *P. aeruginosa* ATTC 27853  *E. coli* (clinical isolate)/  *P. aeruginosa* (Clinical isolate)  *K.pneumoniae*(ATCC 13883)  *P. aeruginosa* ATTC 27853 | NA | 17 |
| *Drechslera halodes* | 6-allyl-5,6-  dihydro-5-hydroxypyran-2-one (**16**) | 12 | 50 | - | *E. coli* | inhibition of DNA synthesis | 90 |
| *Cortinius* sp. | 6-Methylxanthopurpurin-3-O-methyl ether(**18**)  Physcion(**19**)  (1S,3S)-Austrocortilutein(**20**)  (1S,3R)-Austrocortilutein(**21**)  (1S,3S)-Austrocortirubin(**22**)  Torosachrysone(**23**)  Emodin(**24**)  Erythroglaucin(**25**) | - | IC_50_: 31  IC_50_: 2  IC_50_: ≥50  IC_50_: ≥50  IC_50_: ≥50  IC_50_: ≥50  IC_50_: 1.5  IC_50_: ≥50 | - | *P. aeruginosa* | NA | 92 |
| *Pleurotus eous* | Stearic acid(**28**)  Heptadecanoic acid(**29**)  Tartronic acid(**30**) | 5.0±0.00  11.0±0.00  8.0 ±1.00 | 8.8±0.00  3.1±0.00  4.4±0.00 | - | *P. aeruginosa ATCC 29212*  *E. coli ATCC 29995*  *K. pneumoniae CCM 2318* | NA | 97 |

ESBL: Extended Spectrum of β-lactamase; MACI: Multi-drug resistant *Acinetobacter baumanii; P. aeruginosa: Pseudomonas aeruginosa; E. coli: Escherichia coli; K. pneumoniae: Klebsiella pneumoniae; E. faecalis: Enterococcus faecalis;* NA: No data available.
